# Administration of E2 and NS1 siRNAs Inhibit Chikungunya Virus Replication In Vitro and Protects Mice Infected with the Virus

**DOI:** 10.1371/journal.pntd.0002405

**Published:** 2013-09-05

**Authors:** Deepti Parashar, Mandar S. Paingankar, Satyendra Kumar, Mangesh D. Gokhale, A. B. Sudeep, Sapana B. Shinde, V. A. Arankalle

**Affiliations:** National Institute of Virology, Microbial Containment Complex, Sus Road, Pashan, Pune, India; University of Texas Medical Branch, United States of America

## Abstract

**Background:**

Chikungunya virus (CHIKV) has reemerged as a life threatening pathogen and caused large epidemics in several countries. So far, no licensed vaccine or effective antivirals are available and the treatment remains symptomatic. In this context, development of effective and safe prophylactics and therapeutics assumes priority.

**Methods:**

We evaluated the efficacy of the siRNAs against ns1 and E2 genes of CHIKV both *in vitro* and *in vivo*. Four siRNAs each, targeting the E2 (Chik-1 to Chik-4) and ns1 (Chik-5 to Chik-8) genes were designed and evaluated for efficiency in inhibiting CHIKV growth *in vitro* and *in vivo*. Chik-1 and Chik-5 siRNAs were effective in controlling CHIKV replication *in vitro* as assessed by real time PCR, IFA and plaque assay.

**Conclusions:**

CHIKV replication was completely inhibited in the virus-infected mice when administered 72 hours post infection. The combination of Chik-1 and Chik-5 siRNAs exhibited additive effect leading to early and complete inhibition of virus replication. These findings suggest that RNAi capable of inhibiting CHIKV growth might constitute a new therapeutic strategy for controlling CHIKV infection and transmission.

## Author Summary

Despite having immense medical importance, still vaccine, chemoprophylactic, or effective therapeutic measures are not commercially available for chikungunya. Only strict attention to good infection control practices may prevent CHIKV infection. The pathogenic properties of CHIKV necessitate the development of an efficient antiviral therapies. Four siRNAs each, targeting the E2 and ns1 genes of chikungunya were designed and evaluated for their efficiency in inhibiting CHIKV growth in *in vitro* and *in vivo* model systems. Efficiency of these siRNAs in controlling CHIKV replication *in vitro* and *in vivo* was assessed by the real time PCR, IFA and plaque assay. Chik-1 and Chik-5 siRNA ids efficiently inhibited CHIKV replication in the virus-infected Vero-E6 cells and mice. CHIKV replication was completely inhibited in the virus-infected mice when administered 72 hours post infection (p.i.). The combination of Chik-1 and Chik-5 siRNAs exhibited additive effect leading to early and potent inhibition of virus replication. Taken together, these findings suggest the promising efficacy of RNAi ids in silencing sequence-specific genes of CHIKV and might constitute a new therapeutic strategy for controlling the CHIKV infection and transmission.

## Introduction

Chikungunya virus (CHIKV) is a mosquito-transmitted alphavirus belonging to the family *Togaviridae*. CHIKV is responsible for an acute infection, characterized by high fever, arthralgia, myalgia, headache, and rash [1], [2], [3]. Although having immense medical importance, effective vaccine or specific therapy is not commercially available. Currently, strict attention is given to good infection control practices that emphasizes mosquito control program.

RNA interference (RNAi) is the process of sequence-specific, post-transcriptional gene silencing (PTGS) in animals and plants, which is induced by 21- to 23-nucleotide (nt) small interfering RNA (siRNA) that is homologous in sequence to the silenced gene [4], [5], [6]. RNAi not only regulates gene expression in the mammalian cells but also acts as a cellular defense mechanism against the invaders, including the viruses. In recent years, inhibition of specific genes by siRNAs has proven to be a potential therapeutic strategy against viral infection. For instance, inhibition of virus replication and gene expression by directly introducing siRNAs into the cells have been reported for several RNA viruses, including several important human pathogens, such as poliovirus, HIV, Hepatitis, Chandipura and influenza virus [7]–[24]. It has been also shown that alphaviruses such as Semliki Forest virus [7], Venezuelan equine encephalitis [20], O'nyong-nyong virus [14] are susceptible to small interfering RNA action. Recently Dash et al., [22] have demonstrated that introduction of exogenous siRNAs can inhibit replication of CHIKV *in vitro*. The success of this study is limited as siRNAs used against ns3 and E1 genes of CHIKV were shown to reduce replication by 65% by 48 h p.i. and not evaluated in-vivo [22]. Cell clones expressing shRNAs against CHIKV E1 and nsP1 genes showed significant inhibition of CHIKV replication as compared to the scrambled shRNA cell clones and non-transfected cell controls [25].

Alphaviruses contain a linear, positive sense, single stranded RNA genome of approximately 11.8 kb. RNA genome consists of a capped 5′ non-coding region (NCR) and 3′ polyadenylated NCR. The non-structural proteins, nsP1, nsP2, nsP3 and nsP4 are required for the virus replication; the structural proteins, which consist of capsid and envelope proteins (E1, E2, E3 and 6K), are synthesized as polyproteins and are cleaved by capsid autoproteinase and signalases [26]. Given the similarity of the CHIKV genomic structure to those of other alphaviruses, CHIKV is expected to encode spikes on the virion surface that is each formed by three E1–E2 heterodimers where the E1 glycoproteins mediate the fusion and the E2 glycoproteins interact with the host receptor [26],[27],[28]. Nsp1 protein is involved in the RNA synthesis and capping. E2 and ns1 genes are highly conserved in CHIKV strains and are important in the entry and the multiplication in the host cell and, therefore, represent the rational targets for antiviral therapy.

In the current study, based on consensus sequence of CHIKV strains, the siRNA were designed to target the conserved regions in the E2 and ns1 genes of CHIKV. The efficacy of siRNAs targeted against E2 and ns1 genes individually or in combination in inhibiting the replication of CHIKV were evaluated *in vitro* (Vero cells) and *in vivo* (mice).

## Materials and Methods

### Ethics statement

All animals were handled in strict accordance with good animal practice as defined by Institutional Animal Ethics Committee (IAEC). The experiments were done in a biosafety level-2 animal facility at the National Institute of Virology. All animal work was approved by the IAEC. Animal experiments were carried out in strict compliance with Committee for the Purpose of Control and Supervision of Experiment on Animals (CPCSEA) guidelines, India.

### Animals and route of siRNA delivery

Swiss albino and C57BL/6 mice (3–4 wks old; 20–25 grams) were maintained in the BSL-2 facility with controlled temperature (22°C), humidity, and a 12 h light/dark cycle. Mice received the CHIKV via one of three delivery methods: 1) Intra nasal (i.n.) 100 µl, 2) standard intra venous tail vein injection (i.v.) 200 µl, 3) Intra muscular injection (i.m.) 200 µl. siRNA (∼20–25 µg/mouse) mixed with Hiperfect transfection reagent (Qiagen, Germany) and PBS (final volume 200 µl) via i.v. delivery method.

### Vero E6 cells and virus strains

African Green monkey kidney (Vero-E6) cells were maintained in minimum essential medium with 10% fetal bovine serum, 100 U/mL penicillin, 100 µg/mL streptomycin and Neomycine 50 µg/mL. Vero-E6 cells grown under similar conditions were used for the propagation of CHIKV (African genotype, Strain No. 061573; Andhra Pradesh 2006; Accession Number EF027134), Dengue-2 (DENV-2) (Trinidad; TR1751) virus and Chandipura virus (CHPV) (Strain No. 034627; Andhra Pradesh; 2003) stock. CHIKV, DENV-2 and CHPV strains were obtained from virus repository of National Institute of Virology, Pune, India. Virus strains were passaged twice in Vero-E06 cells. Cell supernatants were harvested when 75% of the cells showed cytopathic effect, aliquoted, and stored at −80°C and used throughout the study. The virus stock titers were determined using real time PCR (8.26×10^8^ CHIKV RNA copies/ml) and standard plaque assay (7×10^7^ plaque-forming units/mL).

### siRNA

**Figure 1:**
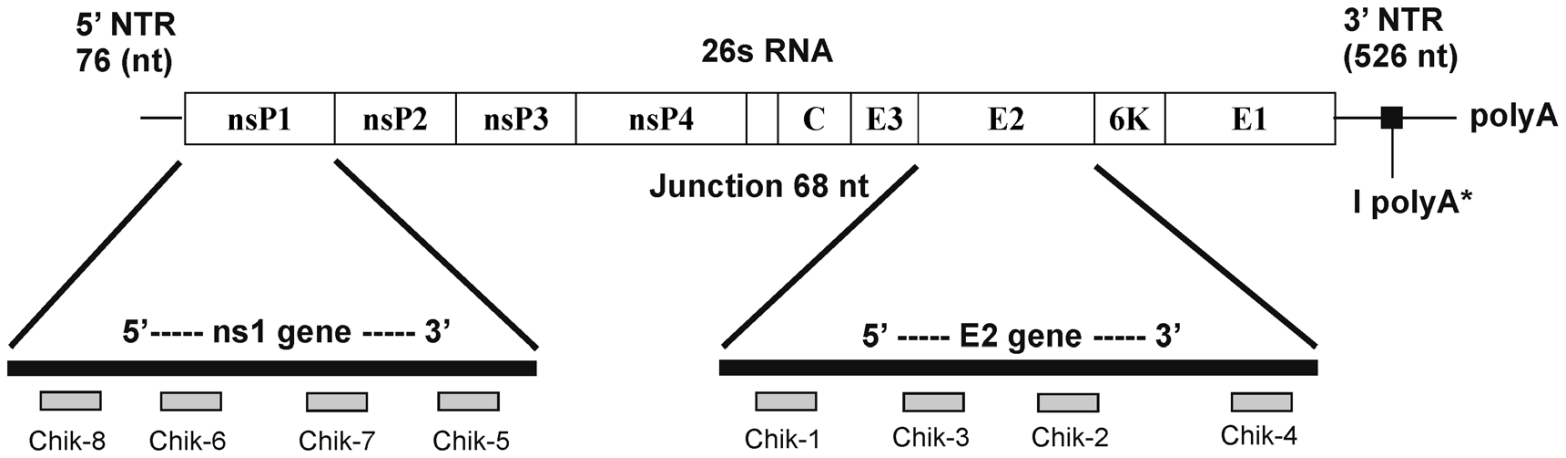
Schematic representation of the sites of the eight siRNA target sequence in CHIKV genome. (Exact location on CHIKV genome is depicted in Table 1.)

CHIKV whole genome sequences were retrieved from GenBank NCBI database (http://www.ncbi.nlm.nih.gov) and consensus sequence was used to design the siRNA. All siRNAs were designed using HP OnGuard siRNA design (Table 1 and Fig. 1). siRNAs were then checked for the homology to all other sequences of the genome using non-redundant sequence database and the homology analysis tool. Four siRNAs each, targeting E2 and ns1 genes were designed and synthesized (Qiagen, Germany) (Table 1, Fig. 1). Negative control siRNA [ncsiRNA; siRNA against Chandipura virus (24) with no significant homology to any known mammalian gene was used as a non-silencing control in all RNAi experiments and were purchased from Qiagen, Germany. Fluorescent labeling of siRNA was performed using the Cy3 Silencer labeling kit (Ambion, USA) and modified as described in the manufacturer's protocol.

**Table 1:** Nucleotide sequences of siRNA designed for CHIKV genes.

No.	Si RNA Name	Location on genome	Location on gene		5′ Sequence 3′
		(nt number)	(nt number)		
**E2 gene**					
1	Chik-1	8574–8591	30–47	Sense	r (GGA CAA CUU CAA UGU CUA U) dTdT
				Antisense	r (AUA GAC AUU GAA GUU GUC C) dTdT
2	Chik-2	8955–8973	411–429	Sense	r (CCA CGA CCC UCC UGU GAU A) dTdT
				Antisense	r (UAU CAC AGG AGG GUC GUG G) dTdG
3	Chik-3	8848–8866	304–322	Sense	r (GGA ACA AUG GGA CAC UUC A) dTdT
				Antisense	r (UGA AGU GUC CCA UUG UUC C) dAdG
4	Chik-4	9386–9404	842–860	Sense	r (CCA CCG UGA CGU ACG GGA A) dTdT
				Antisense	r (UUC CCG UAC GUC ACG GUG G) dGdG
**ns1 gene**					
5	Chik-5	1641–1659	1563–1581	Sense	r (GGU CGA AAU CGA CGU GGA A) dTdT
				Antisense	r (UUC CAC GUC GAU UUC GAC C) dTdG
6	Chik-6	695–713	617–635	Sense	r (GGC UAA GAA CAU AGG AUU A) dTdT
				Antisense	r (UAA UCC UAU GUU CUU AGC C) dTdT
7	Chik-7	1107–1125	1029–1047	Sense	r (CGG CAU CCU UGC UAC AGA A) dTdT
				Antisense	r (UUC UGU AGC AAG GAU GCC G) dGdT
8	Chik-8	290–308	212–230	Sense	r (GGA UGA UGU CGG ACA GGA A) dTdT
				Antisense	r (UUC CUG UCC GAC AUC AUC C) dTdC

### CHIKV infection and transfection

Vero E6 cells were infected with CHIKV (Multiplicity of infection MOI 5. Two h post infection (p.i.), cells were transfected with E2 (Chik-1, Chik-2, Chik-3, Chik-4), ns1 (Chik-5, Chik-6, Chik-7, Chik-8) siRNA and control using the Amaxa Nucleofector device II (Amaxa biosystems). After electroporation, Vero-E6 cells were incubated at 37°C until analyzed for inhibition of CHIKV replication. Cells were harvested at 24, 36 and 48 h p.i. and inhibition of CHIKV replication was determined by quantitative RT-PCR (qRT-PCR), plaque assay and ImmunoFluorescnce Assay (IFA).

### Optimization of siRNA concentration

At two hours p.i. with CHIKV, Vero E6 cells were transfected with Chik-1, Chik-5 and combination of both siRNAs at different concentration (10, 50, 100, 150 and 200 pmol). After 24 h post transfection, total RNA was isolated from the tissue culture supernatant and cells. One step qRT-PCR was carried out to evaluate the inhibitory effect of siRNA.

#### MTT assay

Cytotoxicity tests were performed with Vero E6 cells using an in vitro toxicology assay kit (TOX-1, Sigma) based on the reduction activity of methyl thiazolyl tetrazolium (MTT). Twenty-four hours before transfection, 5×10^3^ cells were seeded in a 96-well plate. The transfection was performed using Hiperfect transfection reagent according to the manufacturer's instructions. The cells were divided into four groups: 1) control group (No Hiperfect and siRNA); 2) Hiperfect group; 3) Chik-1 siRNA group; 4) Chik-5 siRNA group; and 5) Combination of Chik-1+Chik-5 siRNA (Comb-siRNA) group. Transfection of siRNAs was carried out on the following day as described earlier in the CHIKV infection and transfection section. Experiments were conducted with non-transfected or siRNA transfected (100 pmol) cells at 24 and 48 hr post-transfection. 24 hours after the siRNA transfection, the cells of the appropriate groups were subjected to MTT assay (TOX-1, Sigma). Blue formazon, solubilized by adding MTT solubilization solution to the wells, produced by viable cells was quantified in ELISA reader (Biorad, USA) at 570 nm after subtracting the background reading at 650 nm. The data were presented as the percentage of viable cell numbers in the siRNA treated and untreated control wells.

### In vivo inhibition of CHIKV using Chik-1 and Chik-5 siRNA

Swiss albino and C57BL/6 mice (3–4 weeks) were infected with approximately 1×10^6^ pfu of CHIKV (100 µl of 10^7^ pfu/ml; ∼4.5×10^8^ RNA copies/ml) by three routes viz.; i.v., i.n. and i.m. and RNA copies were checked in muscles on 2^nd^, 4^th^, 7^th^ and 14^th^ day p.i.. C57BL/6 mice (4–6 weeks) were infected by CHIKV (100 µl of 10^7^ pfu/ml; ∼4.5×10^8^ RNA copies/ml) and CHIKV RNA copies were measured daily in the blood and muscles by one step qRT-PCR for fourteen days. siRNAs were complexed with HiPerfect™ (QIAGEN, Valencia CA) according to the manufacturer's instructions and ∼25 µg/mouse (1 mg/Kg body wt) was administered i.v. once after 48 or 72 h p.i.. Chik-1 siRNA, Chik-5 siRNA and combination of Chik-1 and Chik-5 siRNAs (Comb-siRNA) were used in different mice groups. Blood (∼200 µl) was collected from siRNA, ncsiRNA, or saline injected mice groups at 0, 1, 2, 3 and 4 day post treatment. CHIKV E3 RNA was quantitated from the sera using qRT-PCR. In C57BL/6 mice, the 72 h time point was chosen for siRNA treatment. Blood and hind limb muscle tissues were harvested from C57BL/6 mice at 0, 1, 2, 3 and 4 day post siRNA injection. The tissues were dissected, weighed, crushed and macerated in liquid nitrogen using mortar pestle, and used for the RNA isolation.

### Quantitative RT-PCR

RNA from the Vero-E6 cells, serum and mice tissues was extracted using QIAmp viral RNA minikit (QIAGEN, Valencia, CA) and trizol (Invitrogen USA) method respectively following the manufacturer's instructions. Viral load in serum and/or tissue samples were determined by absolute quantification using the standard curve method. One step RT-PCR was performed in 25 µl reaction mixture containing 5 µl RNA, 12.5 µl TaqMan One-Step RT-PCR 2× Master Mix, 1 µl 40×(RT+RNAasin) (Applied Biosystems) each 1 µl sense (µM), 1 µl anti-sense (µM) primer and 1 µl TaqMan probe. Primers were selected from the E3 structural protein region. Real-time one step RT-PCR was performed in a 96-well format using 7300 real time PCR system and SDS software V 1.0.2 (Applied Biosystems). The amplification program included: reverse transcription at 48°C for 30 min, initial denaturation at 95°C for 10 min, and 50 cycles of denaturation (95°C for 15 sec) and annealing and extension (60°C for 1 min). After the amplification, a melting curve was acquired to check the specificity of PCR products. A standard curve was generated by the amplification of serial dilutions of in vitro transcribed RNA of CHIKV (10^8^ to 10^2^ serial dilutions). After generation of standard curve, we compared unknowns to the standard curve and extrapolated the value. Viral titers were expressed as RNA copies per ml of serum or per ml of tissue culture suspension or per well or per mg tissue. Detection limit of real time PCR was 10 copies per reaction.

### Plaque assay

Serial dilutions of tissue culture supernatants of infected and siRNA transfected cells were added to a monolayer of Vero E6 cells and the plates were incubated at 37°C for 1 h. After the incubation, the medium was replaced by overlay medium (2× MEM, 2% CMC and 10% FBS (Gibco, USA). The plates were incubated at 37°C for 72 h and the cells were stained with amido black and the plaques were counted.

### Immune sera preparation against the CHIKV genotypes in mice

Groups of 3–4 weeks old swiss albino mice were inoculated intra-peritonealy with the CHIKV African strain (1∶1 vol∶vol mixture of CHIKV and Freund's complete adjuvant) and were maintained under standard laboratory conditions. Two booster doses with CHIKV along with Freund's incomplete adjuvant (1∶1) were administered at weekly intervals. Blood samples were collected at pre and post immunization (7 days after the last dose). IgG antibodies were then purified using protein A column (Merck Biosciences, India) according to the instructions of the manufacturer.

### Immunofluorescence assay

Immunofluorescence assay (IFA) was carried out as described by Sudeep et al. (29). Vero E6 cells were fixed with acetone and blocked with 2% BSA in the phosphate buffered saline (pH 7.4) for 1 h. The cells were incubated with (1∶100) mouse anti CHIKV antibody followed by incubation with FITC-conjugated rabbit anti-mouse (1∶500) antibodies (Invitrogen, USA). Cells were counter stained with Evan's blue for one min. The slides were visualized using fluorescence microscope (Nikon eclipse T2000S and Q capture pro 5.0 software). Negative controls were similarly processed using pre-immune sera.

### Histopathology

Hind limb tissues, excluding the femur bone, were fixed in 4% formaldehyde and were embedded in the paraffin. Thin section of 8 µm size were prepared. Tissues were stained with haematoxylin and eosin. Histopathological evaluation was performed on the muscle tissues of the hind legs from the control (saline injected, ncsiRNA), CHIKV infected (4, 5, 6 and 7 day p.i.), treatment groups (Chik-1 siRNA, Chik-5 siRNA and Comb-siRNA). siRNA treatment was given on third day p.i. and the tissues were harvested at 4, 5, 6 and 7 days p.i. and evaluated for necrosis, inflammation, regeneration, mineralization, fibrosis and the edema. Concurrently IFA was carried out to check the presence of CHIKV. Immunofluorescence assay (IFA) was carried out as described by Sudeep et al. [29]. The slides were incubated with (1∶100) mouse anti CHIKV antibody followed by incubation with Alexa flor 546-conjugated rabbit anti-mouse (1∶200) antibodies (Invitrogen USA). Cells were counter stained with DAPI for 10 seconds. The slides were visualized using fluorescence microscope (Nikon eclipse T2000S and Q capture pro 5.0 software). Negative controls were processed similarly.

### Interferon gene expression analysis employing qPCR

For real-time reverse transcription RT-PCR analysis, hind limb muscle tissues were crushed in liquid nitrogen. RNA was extracted by using TRIzol reagent (Invitrogen) according to the manufacturer's instructions. One step RT-PCR was performed using Quantitect SYBR Green RT PCR kit (Qiagen, Germany). Real-time PCR analysis used the following nucleotide primers: 5′-GGCCGAGGACTTTGATTGCACATT-3′ and 5′- AGGATGGCAAGGGACTTCCTGTAA-3′ for actin beta, 5′- AGGAGGAGTTTGATGGCAACCAGT -3′ and 5′- TCCTCATCCCAAGCAGCAGATGAA-3′ for Interferon alpha (INF-α) (NM_010502), 5′- TGTGGCAATTGAATGGGAGGCTTG-3′ and 5′- TCTCATAGATGGTCAATGCGGCGT -3′ for interferon beta (IFN-β), and 5′-AGCGGCTGACTGAACTCAGATTGT-3′ and 5′- ACTGCTTTCTTTCAGGGACAGCCT-3′ for interferon gamma (IFN-γ) (NM_008337). The 25-µl amplification reaction mixture contained 500 ng total RNA, 0.5 µM each primer pair, 0.25 of reverse transcriptase enzyme and 12.5 µl of 2× SYBR green qPCR Supermix (Qiagen, Germany). Cycling conditions were as follows: one cycle of 50°C for 30 min and one cycle of 95°C for 15 min followed by 45 cycles at 94°C for 15 s, 57°C for 30 s, 72°C for 30 s and 68°C for 15 s. The real-time PCR was performed by using a Rotor-Gene 3000 PCR machine. The data were analyzed with Rotor-Gene real-time analysis software. Each sample was analyzed in duplicate and normalized to actin beta mRNA. Expression changes in interferon alpha, beta and gamma genes in CHIKV infected group, CHIKV infected mice treated with Chik-1, Chik-5 and Comb-siRNA group, and the control mice with Chik-1, Chik-5 and Comb-siRNA treatment were investigated using real time PCR analysis. Mice were mock-infected with CHIKV and treated with siRNA on third day p.i. and then gene expression determined at days 4, 5, 6, and 7 p.i.. Three mice were used for each treatment and each time point.

### Statistical analysis

All data were expressed as mean ± standard deviation. The viral loads were log-transformed for improvement of normality. Statistical significance was determined by Dunnet's test using ANOVA. A value of p<0.05 was considered statistically significant. Fold change was compared using one way ANOVA and the groups were also compared by nonparametric Kruskal-Wallis test for confirmation of results.

## Results

Based on the consensus sequences of CHIKV genomes, four siRNAs each were designed to have an antisense strand complementary to the E2 and ns1 RNA. The sequences and the corresponding genomic positions are shown in Table 1 and Fig. 1 respectively.

### Efficiency of different siRNAs in reducing CHIKV replication in vitro

**Figure 2:**
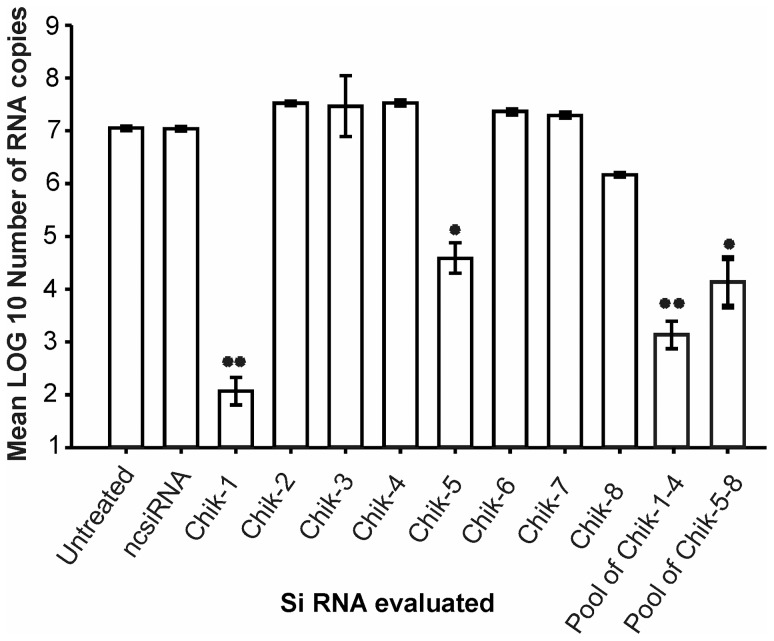
Effect of different siRNAs on CHIKV replication.

**Figure 3:**
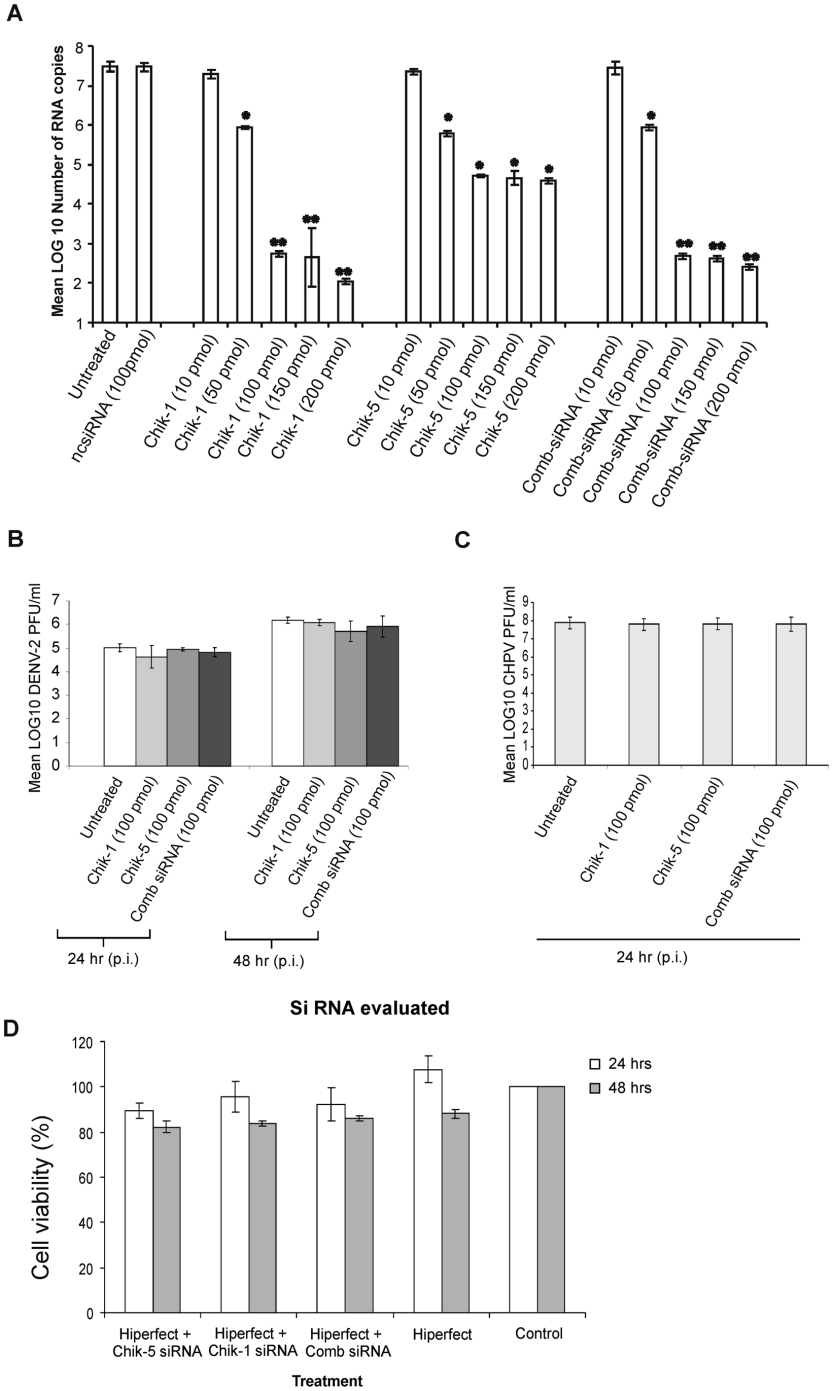
Optimization of siRNA concentration. A) Vero E6 cells were transfected with Chik-1 and Chik-5 siRNAs individually as well as in combination at different concentration (10, 50, 100, 150 and 200 pmol). Total RNA was isolated 24 h after virus infection. Amount of CHIKV RNA was detected by measuring E3 RNA copies by real-time RT-PCR. siRNA Chik-1 and Chik-5 treatment at a concentration of 10 pM failed to reduce CHIKV RNA copies. Whereas all other concentrations of Chik-1 and Chik-5 significantly reduced the viral RNA copies. Values are given as mean log_10_ RNA copies/well ± SD. Significance ANOVA, Dunnett's test: *p<0.05; **p<0.01. **B**) Effect of Chik-1 and Chik-5 siRNAs on DENV-2 growth: Vero-E6 cells were infected with DENV-2 and Chik-1 (100 pmol) CHIK-5 (100 pmol) and Comb-siRNA (100 pmol) transfected after 2 h p.i. and DENV-2 titers were measured by plaque assay. **C**) Effect of Chik-1 and Chik-5 siRNAs on Chandipura virus (CHPV) growth: Vero-E6 cells were infected with CHPV and Chik-1 (100 pmol) CHIK-5 (100 pmol) and Comb-siRNA (100 pmol) transfected after 2 h p.i. and CHPV titers were measured by plaque assay **D**) Effect of siRNA–Hiperfect complex treatment on survival of Vero-E6 cells was accessed by MTT assay. siRNAs were used in 100 pmol concentration.

For the initial comparison of antiviral activity of different siRNAs, Vero-E6 cells were infected with CHIKV and transfected with different siRNAs (Chik-1 to Chik-8) 2 h p.i.. Chik-1 and Chik-5 were the most effective siRNAs, suppressing CHIKV copies by 5 log_10_ (*p*<0.001) and ∼2.5log_10_ (*p*<0.05) RNA copies respectively (Fig. 2). The pool of siRNAs Chik 1–4 (4 log_10_; *p*<0.001) and Chik5–8 (3 log_10_; *p*<0.001) did not increased the CHIKV suppression in Vero E6 cells (Fig. 2). Results obtained with the individual siRNAs and pool of siRNAs indicated that only siRNAs Chik-1 and Chik-5 possessed the antiviral activity against CHIKV. Therefore only Chik-1 and Chik-5 and Comb-siRNA were used for further studies. The reduction in the CHIKV copies by Chik-1 and Chik-5 was initiated at the siRNA concentrations of 50 pmol, and reached a plateau at 100 pmol (Fig. 3 A). Chik-1 and Chik-5 showed sequence dependent inhibition and showed no reduction in the dengue-2 (Fig. 3B) and the Chandipura virus (Fig. 3C) replication in Vero-E6 cells.

### Effect of siRNA treatment on proliferation of VeroE6 cells (MTT assay)

Effect of Chik-1, Chik-5 and Comb-siRNAs (100 pmol) transfection on survival of Vero-E6 cells was assessed by the MTT assay. At 24 h, transfection of Chik-1 siRNA (95.1±6.51), Chik-5 siRNA (89.46±3.19), Comb-siRNA (92.11±7.11) and Hiperfect reagent (95.88±11.47) do not exhibited any significant change in proliferation of Vero E6 cells (Fig. 3D). At 48 h, transfection of Chik-1 siRNA (83.71±9.24), Chik-5 siRNA (82.13±2.71), Comb-siRNA (86.1±1.65) and Hiperfect reagent (88.02±2.58) displayed small reduction in viable cell number (Fig. 3D).

### siRNA stability

Transfected Cyanine 3 dye labeled siRNAs showed signal at 4 h and 6 h whereas at 24 h signal was minimal, but still present compared to control treatment (Supplementary Information Fig. S1). Chik-1 and Chik-5 siRNAs were stable till 24 h.

### Effect of Chik-1 and Chik-5 siRNAs on CHIKV replication

**Figure 4:**
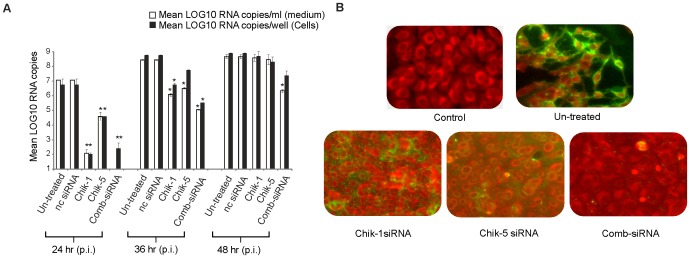
Evaluation of siRNAs directed against CHIKV E2 and ns1. **A**) Quantitative analysis of intra cellular and extra cellular CHIKV RNA copies using real time PCR: Total RNA was isolated at 24, 36 and 48 h after virus infection and E3 RNA copies determined by real-time RT-PCR. Values are given as mean RNA copies/well ± SD for cells and mean RNA copies/ml ± SD for culture medium. Significance ANOVA, Dunnett's test: *p<0.05; **p<0.01; ***p<0.001. **B**) Detection of CHIKV in Vero E6 cells using immuno-fluorescence microscopy: Vero E6 cells seeded in 6 well plates were infected with CHIKV and after 1 h transfected with 100 pmol of the indicated siRNAs. Cells were fixed 48 h later and CHIKV (green) were detected with fluorescein labeled antibodies. Cells were co-stained with Evan's blue (red) to visualize the cell morphology.

Figure 4A depicts the effect of Chik-1, Chik-5 and Comb-siRNAs on the CHIKV replication at different time points. At 24 h p.i., treatment of Chik-1 and Chik-5 siRNAs resulted in the reduction of 5 log_10_ and 3 log_10_ CHIKV RNA copies respectively in cells and the supernatant (Fig. 4A). At 36 h p.i., 3 log_10_ (Chik-1), and 2 log_10_ (Chik-5) reduction in CHIKV RNA copies was observed in tissue culture supernatant whereas 2 log_10_ reduction was recorded in cells with Comb-siRNAs (Fig. 4A). At 48 h p.i., no significant reduction in CHIKV RNA copies was noted in the cells and the supernatant. Overall, the siRNAs directed against E2 gene (Chik-1) were more efficient in inhibiting CHIKV replication than the siRNA directed against ns1 region (Chik-5). We further evaluated the additive advantage of treatment with Comb-siRNAs. In supernatant, 5 log_10_ (p<0.001), 2.5 log_10_ (p<0.05) and 2.5 log_10_ (ANOVA Dunnet's test p<0.05) reduction in CHIKV copies was observed at 24, 36 and 48 h respectively when compared to virus infected control. In cells, 4.5 log_10_ (p<0.001), 3 log_10_ (p<0.05) and 2 log_10_ (p<0.05) reduction was obtained at 24, 36 and 48 h respectively. Importantly, the Comb-siRNA could prolong the inhibitory effect as compared to individual siRNAs (Fig. 4A).

When plaque assay was used as the measure of CHIKV replication, Chik-1 siRNA yielded a reduction of 5 log_10_ at 48 h p.i. (Table 2). Chik-5 reduced 3 log_10_ and Comb-siRNA showed reduction of 3 log_10_ in the virus titer. At 24 and 36 h p.i., cytopathic effects were not observed in the treated cultures where as commencement of cytopathic effects was observed in the untreated control from 24 h p.i. demonstrating the inhibitory effect of the siRNAs. These results were consistent with our real time RT-PCR results and the plaque assay results, IFA also showed the reduction of viral antigen in Chik-1 and Chik-5 siRNAs treated cells (Fig. 4 B).

**Table 2:** Plaque assay showing inhibitory effect of siRNAs on CHIK virus replication in Vero E6 cell line at 48 h.

Material	Virus titer pfu/ml (SD)	Virus inhibition compared to untreated
Untreated	4×10^7^ (1.9×10^7^)	Nil
CHIKV+Chik-1 siRNA	2×10^2^ (1.1×10^2^)	>5 log_10_ **
CHIKV+Chik-5 siRNA	4×10^4^ (1.8×10^4^)	3 log_10_ *
CHIKV+Comb-siRNA	7.5×10^4^ (6.3×10^4^)	∼3 log_10_ *
CHIKV+ncsiRNA	4×10^7^ (1.8×10^7^)	Nil

Values are given as mean pfu/ml (SD).

Significance ANOVA, Dunnett's test:

* *p*<0.05;

** *p*<0.01.

### Swiss albino and C57 BL/6 mice are permissive to CHIKV infection

**Figure 5:**
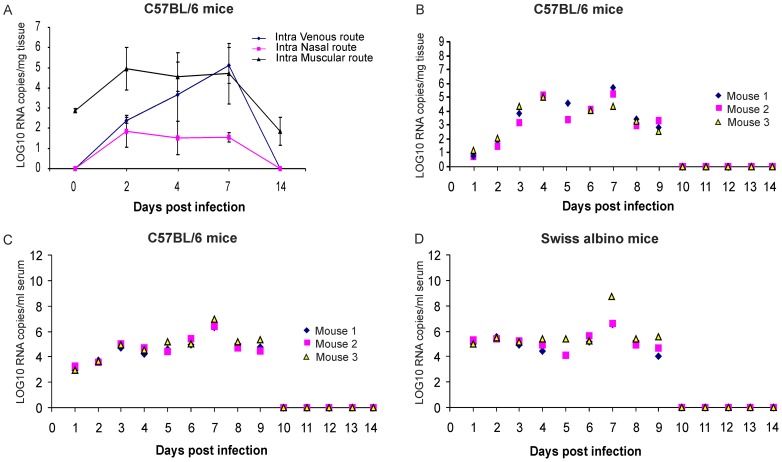
CHIKV replication in C57BL/6 and Swiss albino mice. C57BL/6 mice were infected with CHIKV (1×10^6^ PFU CHKV; 100 µl of 10^7^pfu/ml) by three different routes viz. intra venous, intra nasal and intra muscular. **A**) CHIKV copies were measured in hind limb muscle tissues on 0, 2, 4, 7, and 14 days p.i.. **B**) CHIKV RNA copies in hind limb muscle tissues of C57BL/6 mice on 1–14 days p.i. **C**) CHIKV RNA copies in serum of C57BL/6 mice on 1–14 days **D**) CHIKV RNA copies in serum of swiss albino mice.

As human muscle cells are the target of CHIKV infection, we evaluated the i.m. route along with i.v. and i.n. route for CHIKV infection. Infection by all the three route resulted in CHIKV replication in the thigh muscles (Fig. 5A). CHIKV RNA copies were not detected in uninfected mice at 0, 2, 7 and 14 days p.i.. Mice inoculated with in i.v., i.m. and i.n. routes; CHIKV was not detected in the muscle tissues at 0 day p.i. (Fig. 5A). CHIKV appeared in the muscle tissues by 2 days p.i., persisted till 7 days p.i. and disappeared on 14 days p.i. (Fig. 5A). CHIKV inoculated via i.m. route could be detected in thigh muscle tissues at 2 h p.i. (753±101 CHIKV RNA copies). Therefore i.m. route was not preferred as it was difficult to distinguish newly replicated virus from the virus innoculum. Intra nasally inoculated mice exhibited the lower viral RNA copies in the thigh muscles (Fig. 5A). Since i.m. and i.n. routes were not yielded satisfactory results, therefore we used i.v. route for CHIKV infection.

Swiss albino and C57 BL/6 mice were infected by i.v. route and CHIKV RNA copies were measured in serum and muscle tissues from 1 day p.i. to 14 day p.i.. Infection of adult swiss albino and C57BL/6 mice with 1×10^6^ PFU CHKV (100 µl of 10^7^pfu/ml) CHIKV by i.v. route did not cause mortality. Clinical symptoms such as sluggishness and foot swelling were observed. A definite evidence of the replication of the virus was observed in muscle tissues (Fig. 5B). CHIKV RNA copies were detected in mice serum from 1 day p.i. till 9 days p.i. (Fig. 5 C & D). Viremia in i.v inoculated mice reached a peak by 3 days p.i., with viral loads ranging from 7×10^5^ to 5×10^7^ viral RNA copies/ml (Fig. 5 C & D).

### siRNA inhibits the CHIKV replication in swiss albino mice

**Figure 6:**
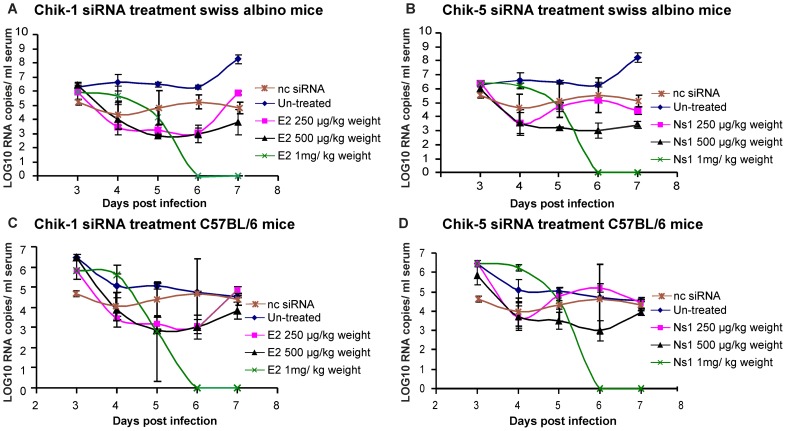
Dose dependent reduction in CHIKV copies/ml serum after injection with siRNA Chik-1 and Chik-5 in swiss albino and C57BL/6 mice infected with CHIKV. Swiss albino and C57BL/6 mice were infected with CHIKV i.v. (1×10^6^ PFU CHKV; 100 µl of 10^7^pfu/ml). After 72 h p.i. swiss albino mice were inoculated i.v. with **A**) Scrambled siRNA (siRNA against chandipura virus), 250 µg, 500 µg and 1 mg/kg body weight Chik-1 siRNA (n = 3 for each treatment and time point), **B**) Scrambled siRNA, 250 µg, 500 µg and 1 mg/kg body weight Chik-5 siRNA (n = 3 for each treatment and time point). After 72 h of p.i. C57BL/6 mice were inoculated i.v. with **C**) Scrambled siRNA, 250 µg, 500 µg and 1 mg/kg body weight Chik-1 siRNA (n = 3 for each treatment and time point), **D**) Scrambled siRNA, 250 µg, 500 µg and 1 mg/kg body weight Chik-5 siRNA (n = 3 for each treatment and time point). At indicated time after injection of siRNA blood was collected from eye and RNA was isolated from serum. CHIKV E3 RNA copies were quantitated using real time RT-PCR. Values are given as LOG10 RNA copies/ml serum.

**Figure 7:**
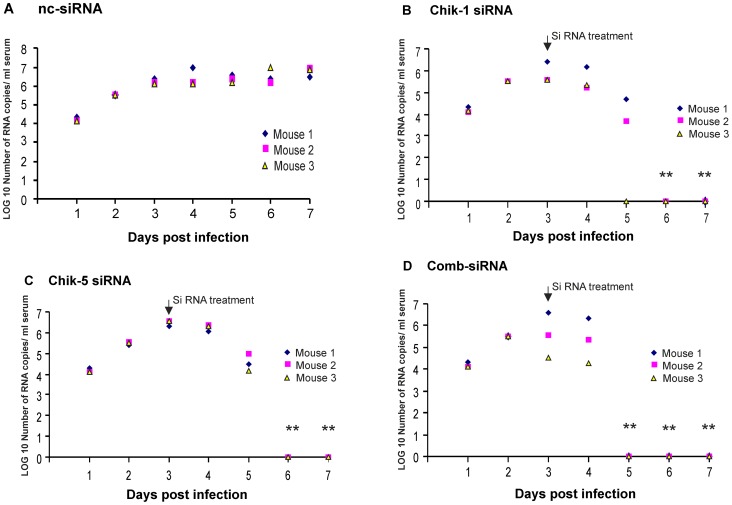
The reduction in CHIKV copies/ml serum after injection with siRNA Chik-1 and Chik-5 in Swiss albino mice infected with CHIKV. Swiss albino mice were infected with CHIKV i.v. (1×10^6^ PFU CHKV; 100 µl of 10^7^pfu/ml). After 72 h of p.i. mice were inoculated i.v. with **A**) ncsiRNA 1 mg/kg body weight **B**) 1 mg/kg body weight Chik-1 (n = 3), **C**) 1 mg/kg body weight Chik-5 (n = 3) and **D**) Comb-siRNA (n = 3) 1 mg/kg body weight each. At indicated time after injection of siRNA blood was collected from eye and RNA was isolated from serum. CHIKV E3 RNA was quantitated using real time RT-PCR. Values are given as LOG10 RNA copies/ml serum. Significance ANOVA, Dunnett's test: *p<0.05; **p<0.01.

To assess whether siRNAs could protect mice from CHIKV infection, groups of CHIKV infected mice (1×10^6^ PFU CHIKV; 100 µl of 10^7^pfu/ml) were administered Chik-1 and Chik-5 siRNAs at 72 h p.i.. Swiss albino mice treated with E2 or ns1 siRNA with 250 µg per kg body weight (∼6 µg/mice) showed ∼3log_10_ inhibition, 500 µg per kg body weight (∼12 µg/mice) showed 3log_10_ inhibition of CHIKV whereas at 1 mg per kg body weight (∼25 µg/mice) siRNA led to 7log_10_ reduction in CHIKV copies (Fig. 6 A & B). Similar results were obtained in C57BL/6 mice (Fig. 6 C & D). We therefore administered 1 mg/kg body weight (∼25 µg/mice) siRNA in subsequent experiments. For all *in-vivo* experiments, Hiperfect reagent was used for delivery of siRNA. Chik-1, Chik-5 and Comb-siRNA administered at 72 h p. i. provided significant reduction in serum viral load as assessed by real time PCR (Fig. 7). At 48 h post siRNA injection, reduction with Chik-1 and Chik-5 was around 2.5 log_10_ (ANOVA Dunnet's test p<0.05) as compared to 0 h and ncsiRNA whereas 100% inhibition (7log_10_) was observed with Comb-siRNA (ANOVA Dunnet's test p<0.01). At 72 h p.i., administration of Chik-1, Chik-5 and Comb-siRNAs showed complete inhibition (7log_10_, ANOVA Dunnet's test p<0.01).

### Inhibition of the CHIKV replication in C57BL/6 mice after treatment of siRNA

**Figure 8:**
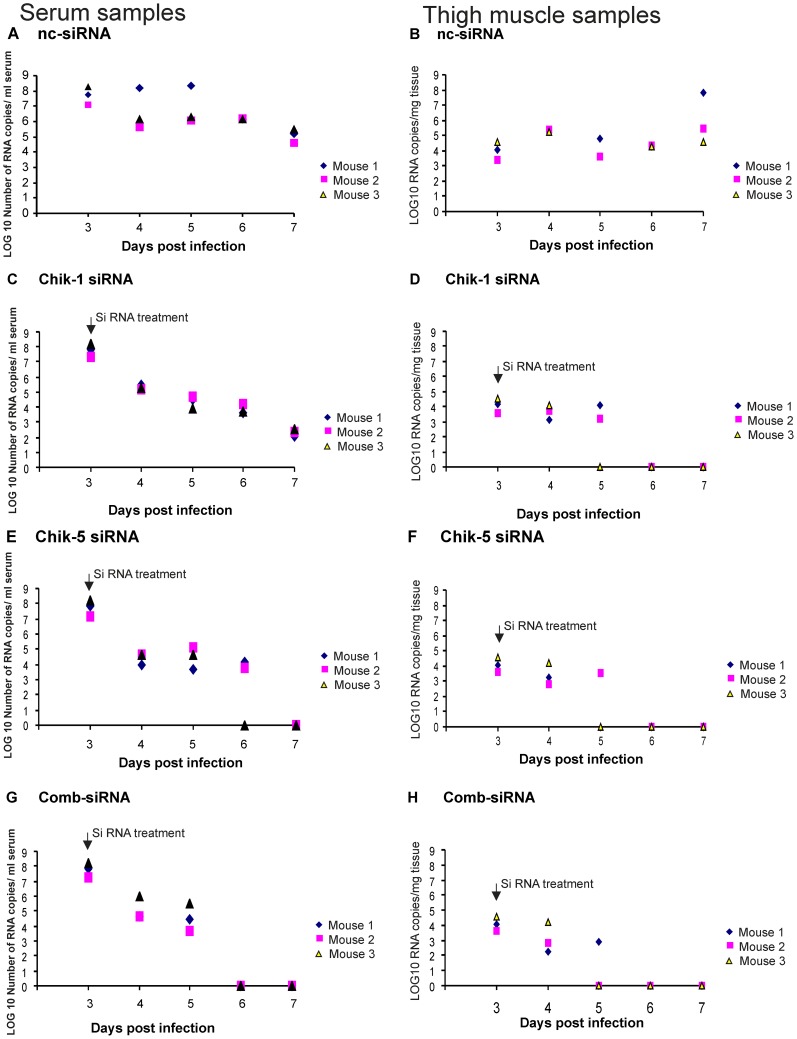
The reduction in CHIKV copies/ml serum after injection with siRNA Chik-1 and Chik-5 in C57BL/6 mice infected with CHIKV. C57BL/6 mice (n = 15) were infected with CHIKV i.v. (1×10^6^ PFU CHKV; 100 µl of 10^7^pfu/ml) and viral RNA copies were checked in serum and muscle tissues. After 72 h p.i. mice were inoculated i.v. with 1 mg/kg body weight nc siRNA (**A** and **B**), 1 mg/kg body weight Chik-1 (n = 15) (**C** and **D**), 1 mg/kg body weight Chik-5 (n = 15) (E and F) and Comb-siRNA (n = 15) 1 mg/kg body weight each (**G** and **H**) and viral RNA copies were checked in serum and muscle tissues at indicated time after injection. CHIKV E3 RNA was quantitated using real time RT-PCR. Values are given as LOG10 RNA copies/ml serum and LOG10 RNA copies/mg of tissue. Significance Dunnett's test: *p<0.05; **p<0.01.

**Figure 9:**
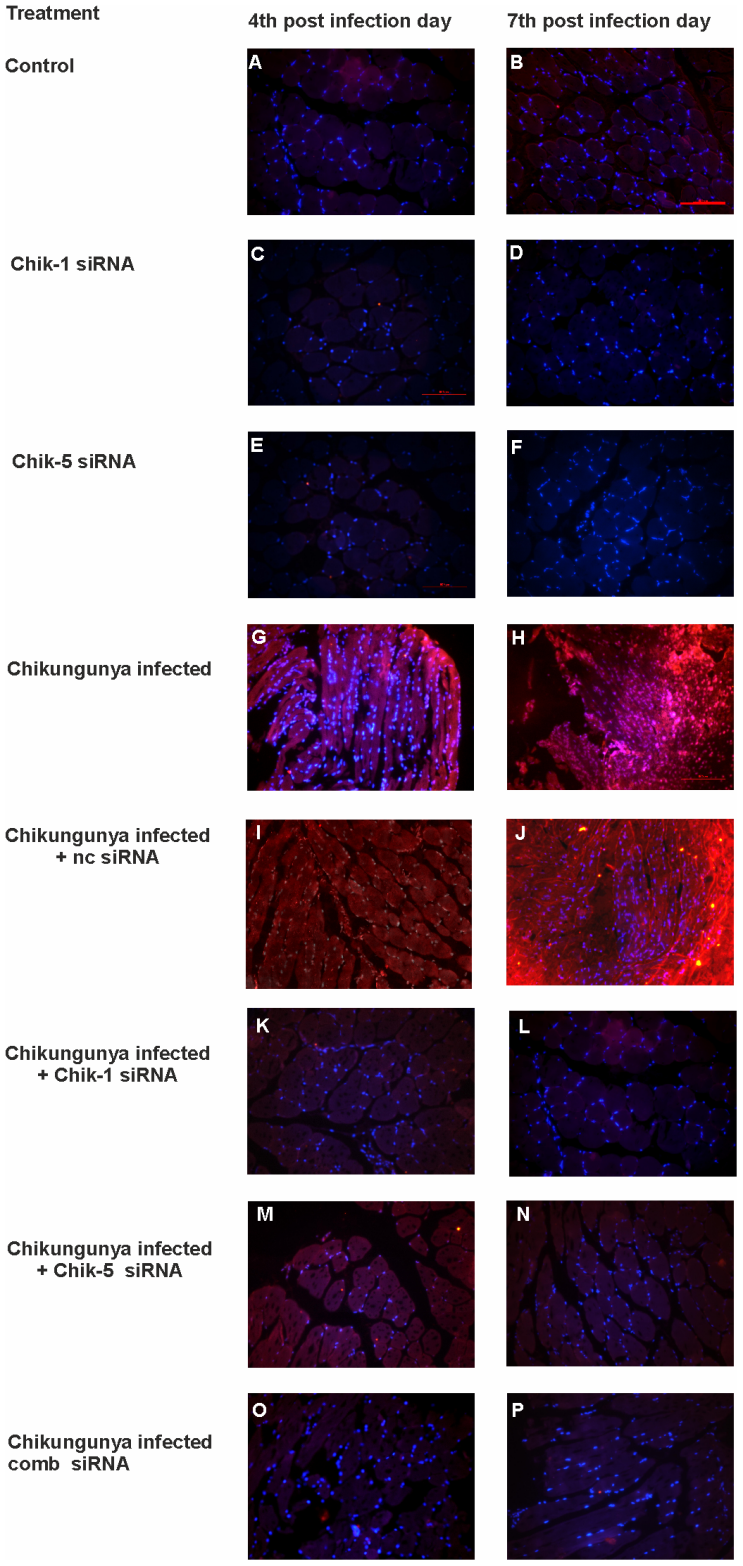
Detection of chikungunya in mouse muscle tissues using Immunofluorescence assay after chikungunya infection and siRNA treatment. C57BL/6 mice were infected with CHIKV i.v. (1×10^6^ PFU CHKV; 100 µl of 10^7^pfu/ml). PBS, ncsiRNA, E2 siRNA and ns1 siRNA injected mice showed absence of chikungunya antigen at 4^th^ and 7^th^ days p.i. (**A**, **B**, **C**, **D**, **E** and **F**). CHIKV infected muscles showed presence of chikungunya antigen (**G** and **H**), ncsiRNA treated CHIKV infected muscles showed presence of chikungunya antigen (**I** and **J**) whereas siRNA treated CHIKV infected mice muscle tissues showed the faint staining of chikungunya antigen (**K**, **L**, **M**, **N**, **O** and **P**) (Magnification ×200).

Chik-1, Chik-5, Comb-siRNA and ncsiRNA administered 72 h p.i. (1×10^6^ PFU CHKV; 100 µl of 10^7^pfu/ml) provided significant reduction in the serum viral load as assessed daily by real time PCR (Fig. 8). At 24 h and 48 h post siRNA treatment, 2.5 log_10_ and 3.5 log_10_ (ANOVA Dunnet's test p<0.05) reduction was recorded for all siRNAs, when compared to ncsiRNA. At 72 h post treatment, reduction with siRNA Chik-1, and Chik-5 was around 3.5 log_10_ (ANOVA Dunnet's test p<0.05) while Comb-siRNA showed 100% inhibition (7log_10_, ANOVA Dunnet's test p<0.01). Importantly, Comb-siRNA produced prolonged inhibitory effect when compared to individual siRNAs. In muscle tissues, CHIKV RNA reached peak by third day p.i., with viral loads ranging from 1×10^4^ to 7×10^5^ viral RNA copies/mg tissue (Fig. 8). At 24 h post-siRNA treatment ∼2.5 log_10_ reduction in CHIKV RNA was noted with all the three siRNAs as compared to ncsiRNA control. At 72 h, all the siRNAs produced 4log_10_ reduction in CHIKV RNA (100% inhibition, ANOVA Dunnet's test p<0.01). Similar results were seen when IFA was used to evaluate the effect of siRNA on CHIKV replication in muscle tissues that corroborated with real time PCR-based data (Fig. 9).

### Histopathological evaluation of mice muscle tissues after CHIKV infection and siRNA treatment

**Figure 10:**
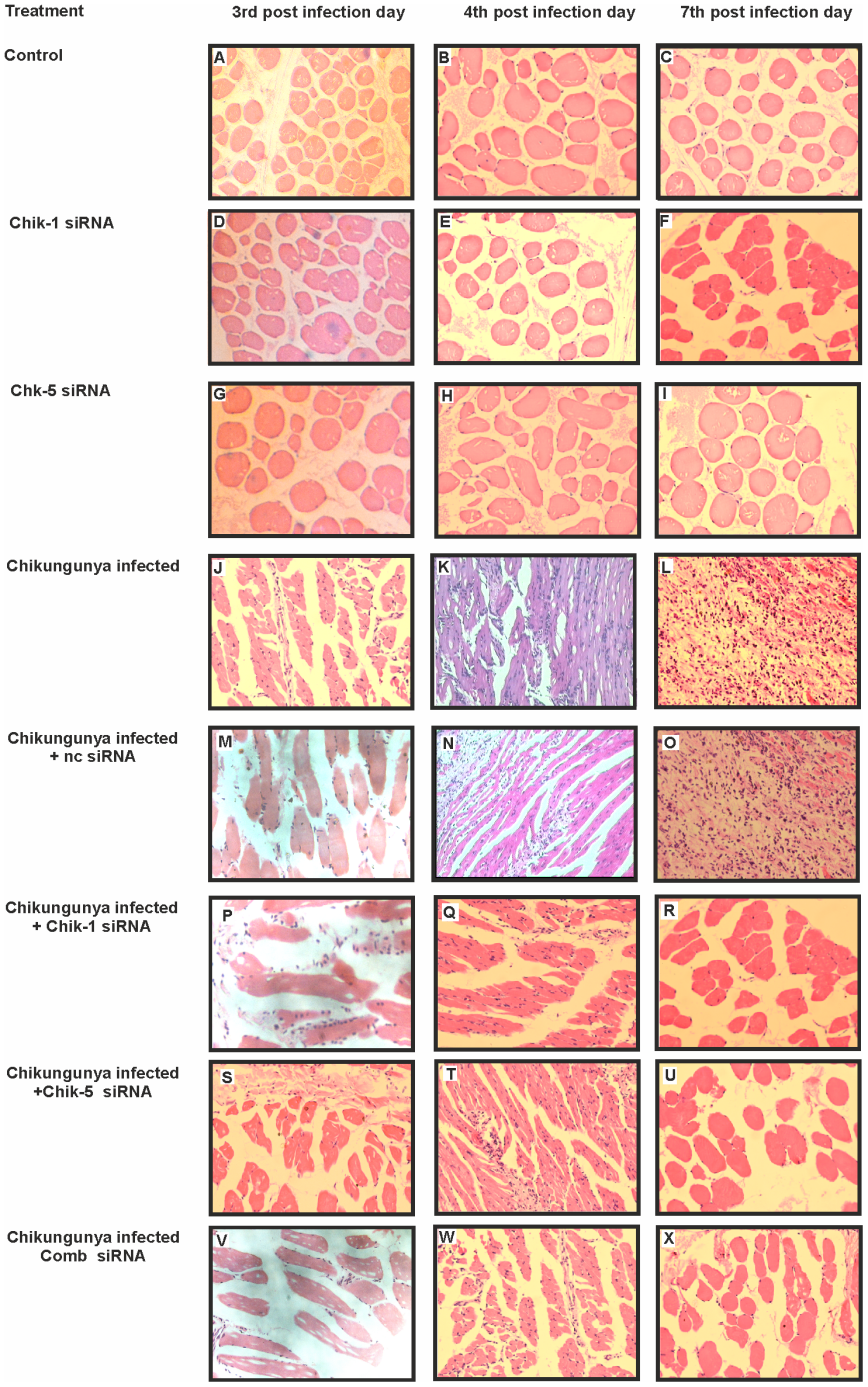
Histopathological changes in mouse muscle tissues after chikungunya infection and siRNA treatment. C57BL/6 mice were infected with CHIKV i.v. (1×10^6^ PFU CHKV; 100 µl of 10^7^pfu/ml). Hematoxylin/eosin-stained tissue sections were screened to investigate the pathological effects of siRNA treatment. PBS injected mice showed normal cellular organization (**A**, **B** and **C**). No significant cellular changes were observed in E2 siRNA treated mice (**D**, **E** and **F**) and ns1 siRNA treated mice (**G**, **H** and **I**). At 3 days p.i. mild inflammation and mild monocyte/macrophage infiltrates were observed (**J**, **M**, **P**, **S** and **V**). At 4 days p.i. and 7 days p.i. CHIKV infected and ncsiRNA treated muscles showed pronounced monocyte/macrophage infiltrates, necrosis and edema (**K**, **L**, N and **O**), whereas siRNA treated CHIKV infected mice muscle tissues showed the regeneration after treatment (**Q**, **R**, **T**, **U**, **W** and **X**) (Magnification ×400).

Having demonstrated that Chik-1 and Chik-5 siRNA treatment significantly reduced the CHIKV titer in serum and muscle tissues, histopathology analysis was performed to determine the inflammation and infiltration in chikungunya infected and siRNAs treated tissues. Histopathological examination of CHIKV infected mice (1×10^6^ PFU CHKV; 100 µl of 10^7^pfu/ml) showed pathological changes such as extensive necrosis, inflammation, pronounced monocyte/macrophage infiltrates and edema (Fig. 10). Such histopathological changes were prevented by systemic treatment either with Chik-1, Chik-5 individually or in Comb-siRNAs. At 3 days p.i., the muscle tissues chikungunya infected mice showed mild inflammation of lymphocytes and monocytes. At 4 days p.i., chikungunya infected mice muscle tissues showed moderate inflammation of lymphocytes and monocytes, focal edema and focal necrosis whereas siRNA treated mice muscle tissues showed only mild inflammation. At 7 days p.i., extensive muscular necrosis with dense inflammation of lymphocytes and monocytes was observed in CHIKV infected and ncsiRNA treatment mice. On other hand, siRNA treatment preserved the morphological integrity of the muscle tissues with regeneration (Fig. 10). The muscle tissues from control mice infected with saline showed no pathological changes such as necrosis, edema, inflammation and infiltration of polymorphs (Fig. 10).

### Expression levels of interferon genes after siRNA treatment

We tested if inhibition of CHIKV replication in mice was indeed sequence dependent and not because of non-specific antiviral interferon response. In the absence of CHIKV, Chik-1, Chik-5 and Comb-siRNA treatment did not significantly induced the α, β and γ interferon mRNA expression in hind limb muscle tissues (Table 3; Kruksal Wallis p>0.05). Similarly, siRNA treatment of CHIKV-infected mice did not show significant elevations in α, β and γ interferon gene expression in the hind limb muscle tissues (Table 3; Kruksal Wallis p>0.05). These results suggest that siRNA mediated reduction in CHIKV replication is sequence specific without any deleterious effect on the host.

**Table 3:** Interferon response in hind limb muscle tissue of C57BL/6 mice after treatment with siRNAs.

Post infection period	Treatment			Relative fold change compared to control		
	Chikungunya infection	Chik-5 siRNA	Chik-1 siRNA	Interferon α	Interferon β	Interferon γ
				Mean (SD)	Mean (SD)	Mean (SD)
**4 days p.i.**						
(n = 3)	+			0.94 (0.88)	2.03 (1.31)	25.22 (21.12)
(n = 3)	+	+		2.83 (2.19)	1.81 (1.61)	8.25 (5.06)
(n = 3)	+		+	2.06 (1.77)	2.69 (2.21)	18.00 (16.80)
(n = 3)	+	+	+	1.21 (0.80)	3.99 (3.82)	4.55 (1.23)*
(n = 3)		+		1.54 (1.21)	5.18 (3.17)	1.09 (0.60)
(n = 3)			+	0.95 (0.66)	4.76 (3.23)	1.08 (0.64)
(n = 3)		+	+	2.19 (0.46)	6.00 (4.55)	7.09 (5.18)
**5 days p.i.**						
(n = 3)	+			0.11 (0.05)	0.41 (0.33)	371.73 (236.04)
(n = 3)	+	+		1.88 (1.48)	1.71 (1.54)	19.44 (11.93)
(n = 3)	+		+	4.65 (2.08)	4.74 (4.14)	44.38 (41.70)
(n = 3)	+	+	+	0.81 (0.30)	3.59 (3.49)	163.09 (65.36)*
(n = 3)		+		1.22 (0.53)	4.69 (1.06)	3.31 (1.91)
(n = 3)			+	0.60 (0.44)	6.54 (0.74)	0.97 (0.06)
(n = 3)		+	+	3.38 (2.63)	5.55 (4.75)	1.49 (2.00)
**6 days p.i.**						
(n = 3)	+			2.15 (2.05)	0.42 (0.27)	9.37 (5.87)
(n = 3)	+	+		2.50 (1.27)	3.61 (3.06)	6.06 (4.64)
(n = 3)	+		+	14.27 (9.47)	1.54 (1.35)	50.74 (42.74)
(n = 3)	+	+	+	5.15 (4.80)	4.85 (2.20)	344.23 (294.86)
(n = 3)		+		0.45 (0.10)	4.89 (3.59)	1.10 (0.74)
(n = 3)			+	0.62 (0.42)	5.71 (1.40)	0.68 (0.65)
(n = 3)		+	+	3.67 (3.47)	6.16 (5.59)	0.78 (0.64)
**7 days p.i.**						
(n = 3)	+			0.67 (0.56)	2.23 (1.90)	4071.74 (705.74)
(n = 3)	+	+		2.36 (1.98)	0.54 (0.05)	7.51 (1.50)
(n = 3)	+		+	18.10 (13.29)	6.44 (5.12)	2.15 (1.87)
(n = 3)	+	+	+	1.32 (1.31)	4.83 (0.33)	119.31 (91.42)
(n = 3)		+		0.58 (0.07)	2.67 (0.18)	1.13 (0.17)
(n = 3)			+	0.52 (0.02)	6.35 (3.68)	2.39 (0.28)
(n = 3)		+	+	2.17 (1.53)	5.74 (5.13)	27.38 (20.14)

Mice were treated with siRNA and gene expression changes in interferon alpha, beta and gamma was monitored in hind limb muscle tissues (n = 3 for each treatment) at 24 h, 48 h, 72 h and 96 h p.i.. Results expressed as 2^−ΔΔCT^ were reported as mean ± standard deviation and were analyzed using Kruksal Wallis test.

* p<0.05 significantly different gene expression change as compared to chikungunya infected mice of respective time point.

Taken together, this first in vivo experiment clearly revealed that siRNA therapy is effective *in vivo* by reducing the clinical symptoms in the challenge-infected animals and was capable of significantly reducing virus replication in the serum and muscles.

## Discussion

This study for the first time clearly shows the efficacy of ns1 and E2 siRNAs in combination, in inhibiting CHIKV replication in mice infected with the CHIKV (1×10^6^ PFU CHIKV; 100 µl of 10^7^pfu/ml, ANOVA Dunnet's test p<0.01). Importantly, a single i.v. inoculation of the siRNAs, 72 h after CHIKV infection could completely inhibit viral replication as evidenced by the absence of viral RNA in the muscles and serum. Though attractive, the therapeutic potential of siRNAs in treating viral diseases has been limited primarily because of the failure when evaluated in animal models and the absence of appropriate delivery systems. However, the success of *in-vivo* use of siRNAs in viral infection is noteworthy [17],[23],[24].

E2 and ns1 genes were chosen as the target genes because of the critical roles in viral replication. E2 and ns1 genes are highly conserved in CHIKV strains. CHIKV is expected to encode spikes on the virion surface, each formed by three E1–E2 heterodimers where the E1 glycoproteins mediate fusion and the E2 glycoproteins interact with the host receptor [26],[27],[28],[30]. Together with nsP4, nsP1 is expected to catalyze the initiation of negative strand RNA synthesis. Nsp1 protein is also involved in methylation and capping of positive RNA [31]. Indeed, CHIKV nsp1, a 535 amino acid long protein contains consensus sequence at the N terminal region which is characteristic of Alphaviruses. Elimination of nsp1 abolishes the ability of CHIKV to replicate. We evaluated four different siRNAs each against ns1 and E2 genes when administered two h p.i. *in vitro*. Of these, Chik-1 (siRNA for E2 gene) and Chik-5 (siRNA for ns1 gene) were most efficient in inhibiting CHIKV replication as assessed by multiple parameters such as real time PCR, plaque assay and IFA (Fig. 2, Fig. 3, Fig. 4, Table 2). The work described here utilizes targets against viral RNA sequences that are conserved, and invariant between different strains. The target sites of Chik-1 and Chik-5 siRNA are 100% conserved in all sequenced CHIKV genome. As compared to individual Chik-1 and Chik-5, the pool of Chik-1–4 and Chik5–8 siRNAs does not have any additive effect on the CHIKV inhibition. The combination therapy of Chik-1 and Chik-5 siRNAs provided additive effect and was found to be most effective as compared to individual siRNA in inhibiting CHIKV replication even at later time points. Thus, simultaneous silencing of multiple genes of CHIKV appeared to be better strategy for preventing viral replication. Recently Dash et al., [22] have used siRNA against ns3 and E1 genes of CHIKV and showed reduction in CHIKV replication by 65% by 48 h p.i. and not evaluated *in-vivo* [22]. *In vitro* studies employing Chik-1, Chik-5 and Comb-siRNAs showed more than 90% reduction in CHIKV replication with all three formulations, whereas complete inhibition of CHIKV replication was observed in *in vivo* studies. The observed efficient inhibition of CHIKV might be due to targeting of conserved sites, route of delivery or combination of both.

The next step was to evaluate these siRNAs in a suitable animal model. Recently C57BL/6 mice were established as susceptible murine models for CHIKV infection [32],[33]. In the absence of an immunocompetent mouse model replicating clinical symptoms in humans, we used Swiss albino and C57BL/6 adult mice for the evaluation of siRNAs. Though mice strains showed mild clinical symptoms (sluggishness and swelling of foot), replication of CHIKV was evident and the effect of siRNAs on CHIKV replication could be assessed employing several parameters (Fig. 5, Fig. 6, Fig. 7 and Fig. 8). SiRNAs were stable in Vero-E6 cells till 24 h post transfection (Fig. S1). We could not study the stability of siRNAs in mice because of low signal of cyanine 3 dye labeled siRNAs. However, it may be noted that previous studies have demonstrated uptake of siRNAs in the liver, kidney, lung endothelium and jejunum using standard i.v. injection of siRNA [34] [35] [36]. Despite the weaker siRNA uptake with standard intra venous administration, earlier reports suggest that this technique is still effective and may offer a potential route for systemic therapeutic use. Standard i.v. administration of siRNA efficiently delivered the siRNAs to various organs and resulted in efficient reduction of CHIKV titer.

Although chikungunya viremia has not been extensively studied in humans, studies on non human primates and mice suggest viremia of short duration, with viral loads ranging from 1×10^3^ to 1.2×10^10^ viral RNA copies/ml [33],[37],[38],[39],[40]. It has been demonstrated that CHIKV can persist for longer time in animal models [37],[40]. The findings of Morrison et al., [40] and Labadie et al., [37] indicate that though CHIKV is readily cleared from most tissues after the acute stage of infection, CHIKV RNA persists in joint tissues for at least 3–4 weeks after inoculation. In monkey model a clear relationship has been demonstrated between the inoculation dose and the period and magnitude of the viremia [37]. In current study we used the high dose of CHIKV (1×10^6^ PFU) which might resulted in longer persistence of CHIKV RNA (7–8 days post inoculation). The results obtained in this study are consistent with findings of Morrison et al., [40] and Labadie et al., [37]. It has been demonstrated that CHIKV actively suppresses STAT activation by both type I and type II interferon [37],[41]. In current study moderate suppression in Interferon α and Interferon β was observed in CHIKV infected mice. It is possible that CHIKV persistence observed in current study might be the combined effect of high dose of CHIKV, route of inoculation, active evasion of host innate or adaptive immune responses by the CHIKV. It will be interesting to evaluate the mechanisms involved in CHIKV persistence in C57BL/6 mice.

Similar to *in-vitro* studies, Comb-siRNA was more efficient than the individual components *in-vivo* studies also. Important point is that the siRNA was administered 48 or 72 h p.i. suggesting utility in CHIKV-infected hosts. CHIKV was detected in muscle tissues of infected mice inducing pathological changes such as severe necrosis and dense infiltration of monocytes and lymphocytes (Fig. 9 and 10). On the contrary, ∼1 day after siRNA treatment, mild inflammation and infiltration of monocytes was observed while after ∼4 days, regeneration and intact muscle morphology with no evidence of inflammation was recorded (Fig. 10). These results clearly demonstrate the therapeutic effect of siRNAs, especially Comb-siRNA, in virus-infected mice. Even a single dose administered 3 days p.i. could protect mice suggesting ability of the siRNAs in treating an established virus infection.

Under certain circumstances, siRNAs can induce the interferon (IFN) pathway and trigger inflammation [42],[43],[44]. It has been suggested that canonical small interfering RNA (siRNA) duplexes are potent activators of the mammalian innate immune system [42],[43],[44]. Synthetic siRNA in delivery vehicles that facilitate cellular uptake can induce high levels of inflammatory cytokines and interferons after systemic administration in mammals and in primary human blood cell cultures [42],[43]. To differentiate the modes of protection offered by siRNAs, we determined expression levels of interferon α, β and γ interferon genes in the muscle tissues of different mice groups (Table 3). siRNAs alone did not induce significant induction of interferon genes; CHIKV-infected, siRNA treated mice did not show siRNA-induced interferon gene expression when compared to the virus infected mice. These results revealed that the observed inhibition of CHIKV replication was mainly because of characteristic activity of siRNAs.

In conclusion, Comb-siRNAs (E2 and ns1 genes) described by us provide an excellent therapeutic agent for chikungunya and should be further assessed in non-human primates. Need for a proper delivery system for use in humans remains an important issue.

## Supporting Information

Figure S1 **Stability of siRNAs.** Cyanine 3 dye labelled Chik-1 and Chik-5 were transfected in Vero-E6 cells using Hiperfect reagent (Qiagen, Germany). After 4 h and 24 h cyanine 3 dye fluorescence signal was detected using fluorescence microscope (Nikon eclipse T2000S and Q capture pro 5.0 software).
(DOC)
