# Supplementary material for: Administration of E2 and NS1 siRNAs Inhibit Chikungunya Virus Replication In Vitro and Protects Mice Infected with the Virus
Source: PLoS Negl Trop Dis. 2013 Sep 5;7(9):e2405. doi: 10.1371/journal.pntd.0002405 (PMC3764232; doi:10.1371/journal.pntd.0002405)

**Fig.S1: Stability of siRNAs** Cy3 labelled Chik-1 and Chik-5 were transfected in Vero E-06 cells using Hiperfect (Qiagen, Germany) reagent. After 4h and 24h cyanine 3 dye fluorescence signal was detected using fluorescence microscope (Nikon eclipse T2000S and Q capture pro 5.0 software).


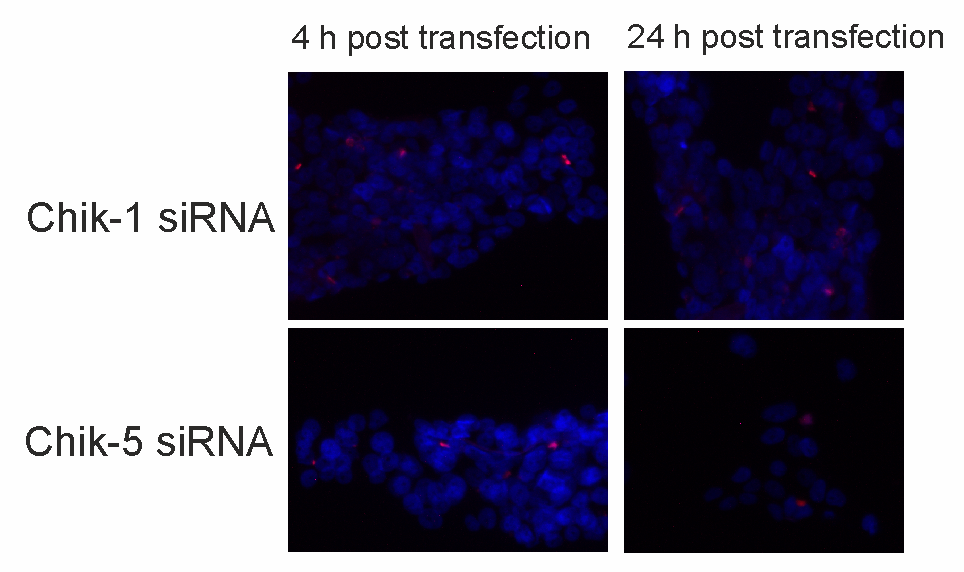

Supplement: Figure S1 — Stability of siRNAs. Cyanine 3 dye labelled Chik-1 and Chik-5 were transfected in Vero-E6 cells using Hiperfect reagent (Qiagen, Germany). After 4 h and 24 h cyanine 3 dye fluorescence signal was detected using fluorescence microscope (Nikon eclipse T2000S and Q capture pro 5.0 software). (DOC) [file pntd.0002405.s001.doc]
